# Investigation of the Microstructure and Compressibility of Biodegradable Fe-Mn-Cu/W/Co Nanostructured Alloy Powders Synthesized by Mechanical Alloying

**DOI:** 10.3390/ma14113088

**Published:** 2021-06-04

**Authors:** Hany R. Ammar, Subbarayan Sivasankaran, Abdulaziz S. Alaboodi

**Affiliations:** 1 Department of Mechanical Engineering, College of Engineering, Qassim University, Buraydah 51452, Saudi Arabia; sivasankaran@qec.edu.sa (S.S.); alaboodi@qec.edu.sa (A.S.A.); 2 Metallurgical and Materials Engineering Department, Faculty of Petroleum and Mining Engineering, Suez University, Suez 43511, Egypt

**Keywords:** Fe-Mn (Cu-W-Co) alloy, biodegradable materials, microstructure, compressibility

## Abstract

In this research work, the nanostructured Fe-Mn (BM0), Fe-Mn-Cu (BM1), Fe-Mn-W (BM2), and Fe-Mn-Co (BM3) biodegradable alloys were successfully synthesized using mechanical alloying. The microstructure of the synthesized alloys was examined using XRD, SEM equipped with EDS, and HRTEM techniques. The results obtained based on these techniques confirmed the development of nanostructured BM0, BM1, BM2, and BM3 alloys and homogenous solid solutions with an even elemental dispersion. The compressibility of the synthesized alloys was investigated experimentally and empirically in the as-milled conditions and after applying a stress relief treatment (150 °C for 1 h). The load applied for compaction experiments ranged from 25–1100 MPa with a rate of 1 mm/min. According to the experimentation performed in the current study, the relative density of the as-milled BM0, BM1, BM2, and BM3 alloys was 72.90% and 71.64%, 72.32%, and 72.03%, respectively. After applying the stress relief treatment, the density was observed to increase to 75.23%, 77.10%, 72.65%, and 72.86% for BM0-S, BM1-S, BM2-S and BM3-S samples, respectively. A number of compaction models were tested to identify the optimum models for predicting the compressibility behavior of nanostructured Fe-Mn, Fe-Mn-Cu, Fe-Mn-W, and Fe-Mn-Co alloys in the as-milled and stress-relieved conditions.

## 1. Introduction

Recently, biodegradable metals and alloys are being widely used as load-bearing temporary implants in medical applications. The function of these materials is to mechanically support a treated tissue during a curative course and then degrade gradually without any remaining implantation; therefore, the recovery process can be completed without any further surgical treatments [1]. Biodegradable materials have required high strength and high degradation rate in addition to the compatibility. Accordingly, several metals are being used as biomaterials such as Fe, Mg, Zn, and their alloys. Iron and its alloys are promising biodegradable materials, for instance stent implementation, due to their acceptable strength and stiffness; however, the low corrosion rate of biodegradable iron alloys is one of the major concerns of these materials [2,3,4]. Several studies were conducted to examine the mechanical behavior and the corrosion behavior of iron-based implant materials [5,6,7,8]. These studies focused on investigating the influence of alloy production techniques and the effect of adding various alloying elements such as manganese, silicon, sulfur, and carbon in order to examine the mechanical behavior and degradability of these alloys [9,10,11,12]. Bagha et al. [13] discussed the degradability of iron based biodegradable implant materials that were produced by means of ball milling and spark-plasma sintering techniques. The authors have reported that the corrosion rate of these materials was accelerated using appropriate addition of Mn and Ag, as alloying elements. The examined alloy displayed high shear strength of 420 MPa and produced the average strain of 66%. Sotoudehbagha et al. [14] have studied various synthesizing method to produce iron-based implant materials and further, the authors have varied different elemental composition. The authors have produced novel iron-based materials consisting of 30 weight fraction of manganese and around 3 weight fraction of silver using ball milling technique. The results revealed that the alloy contained 3 wt.% silver has exhibited the best properties as compared with the other alloys. The investigated properties include the shear strength, micro-hardness, corrosion rate, and relative density. On the other hand, they have reported that the optimal cytotoxicity and antibacterial behavior were obtained when adding 1 wt.% silver. Bagha et al. [15] studied the mechanical behavior, corrosion rate, and biocompatibility of a nanocrystalline Fe-35 wt.% Mn biodegradable alloy. The alloy was synthesized by means of ball milling technique and consolidated by traditional cold pressing and sintering. The milled alloy has exhibited higher hardness, enhanced compression strength, lower rate of corrosion, and improved cell adhesion, as compared to un-milled samples. Safaie et al. [16] successfully synthesized a solid solution of nanostructured iron-based alloy mixed with 30 weight fraction of manganese using ball milling technique. The authors have used the ball milling parameters of 10 h milling time, and 30:1 ball-to-powder ratio. Sikora-Jasinska et al. [17] developed Fe-Mg_2_Si composite for the applications of biodegradable implants with improved mechanical characteristics and increased the corrosion resistance. The produced composite of Fe-Mg_2_Si were synthesized using ball milling technique and then consolidated by hot rolling. The results explained that the incorporation of one weight fraction of magnesium silicide (Mg_2_Si) in the iron matrix was improved the corrosion resistance. Mouzou et al. [18] investigated the biodegradability performance of Fe-20Mn-1.2C alloy in various corrosive solutions. The alloys were fabricated industrially by casting process followed by hot rolling. The highest degradation rate was reported when using commercial Hank’s solution as a corrosion media while the lowest rate was found when using a corrosive media of Dulbecco’s modified solution. Mandal et al. [19] developed a novel Fe-Mn-Cu alloy for fracture fixation with improved anti-microbial behavior. The authors have developed different compositions of iron alloyed with various percentage of manganese and copper in which the maximum manganese content was 35 wt.% and the copper content was 10%. These alloys were manufactured using high-energy mechanical alloying process followed by compaction and sintering. It was reported that adding up to 10 wt.% Cu resulted in increasing the corrosion rate with 6 times more than the base alloy. Furthermore, increasing the Cu-level up to 5 wt.% resulted in a significant increase in the hardness of the investigated alloys. Additionally, increasing Cu-content led to improved anti-microbial properties. Faruk Mert [20] has investigated the tribological behavior of magnesium-based biodegradable alloy (AZ1B hot rolled alloy) for implant applications. The sliding wear test was conducted using pin-on-disc apparatus in which the author has varied the load starting from 10 N to 80 N, the sliding velocity varied from 0.25 m/s to 2.5 m/s, and the maximum sliding distance was 5000 m. Finally, the author has found that the hot rolled AZ1B magnesium-based implant materials were produced more wear resistant. Powder metallurgy (P/M) process is a common one by which several metallic products are being manufactured commercially for aircraft, space craft, automotive, and structural components [21]. Achievement of near net shape and making of complex products are the main features of P/M process [22]. Study of input metal powders before processing is very important as the powder compressibility influence the mechanical performance of P/M products [23]. Further, the compressibility of alloy powders is considered as a substantial stage in industrial application when fabricating P/M products [24]. The compaction behavior of alloy powders is affected by several factors such as the composition, size and shape of the powders, compaction pressure, compaction rate, density of powders, and flow properties of the powders [25,26,27]. Accordingly, the compressibility of the synthesized alloys was investigated for the processed powders in the as-milled conditions and after applying a stress relief treatment.

Fe-Mn-based alloys are promising candidates for biodegradable applications. The biodegradable materials require high strength, acceptable ductility, and high rate of degradation. Several alloying elements were used to improve the mechanical properties and increase the degradation rate [28,29]. The additions of Cu, W, and Co, as alloying elements to an Fe-Mn system have a strong potential to improve the mechanical properties and degradation rate of these biodegradable materials. The present study was conducted for developing and investigating the behavior of Fe-Mn-Cu/W/Co nanostructured biodegradable materials. The purpose of the present study includes: (i) synthesizing Fe-Mn, Fe-Mn-Cu, Fe-Mn-W, and Fe-Mn-Co nanostructured biodegradable alloys by mechanical alloying; (ii) microstructure investigation of the processed alloys using XRD, HRSEM equipped with EDS, and HRTEM; (iii) experimental investigation of the compressibility of the processed alloys in the as-milled condition and after applying stress relief treatment to evaluate the attained relative density of the examined alloys; and (iv) empirical investigation of the compaction behavior by a number of linear and non-linear models for expecting the green density of the produced nanocrystalline alloys in the as-milled and stress relieved conditions.

## 2. Materials and Methods

### 2.1. Synthesis of Fe-Mn, Fe-Mn-Cu, Fe-Mn-W, and Fe-Mn-Co Alloys

Fe, Mn, Cu, W, and Co elemental powders were used for mechanical alloying (MA) process. These powders displayed a high purity more than 99.9% and an average particle size less than 44 μm. Table 1 shows the composition in atomic (at.%) and weight (wt.%) percent of the produced Fe-Mn, Fe-Mn-Cu, Fe-Mn-W, and Fe-Mn-Co alloys. The laboratory ball mill of the type Pulverisette 5/2 classic line was used in performing the mechanical alloying of the starting powders, where two tungsten carbide (WC) vails with a capacity of 250 mL (for each vail) and WC balls with 10 mm diameter were used in the present study. The mixed elemental powders were subjected to MA for five hours with a speed of 300 rpm and BPR of 10:1. The MA was accomplished in a liquid grinding medium of toluene (purity >99.9%) to avoid cold welding and oxidization of the mechanically alloyed (MAed) powders [21,30]. MA was completed based on the frequent milling cycles that comprises 15 min milling in the forward direction followed by 15 min pause and thereafter 15 min milling in the reverse direction followed by a pause of 15 min; this milling cycle was repetitive to achieve continuous MA process with the least heat buildup inside the vails. Some quantity of synthesized powders was stress relieved at 150 °C for 1 h under vacuum using R120/500/13 model tube furnace (Nabertherm, Lilienthal, Germany). The notation used in the samples are BM0 (Fe-65, Mn-35, at.%), BM1 (Fe-65, Mn-32, Cu-3, at.%), BM2 (Fe-65, Mn-32, W-3, at.%), and BM3 (Fe-65, Mn-32, Co-3, at.%).

### 2.2. Microstructural Characterization of the Processed Fe-Mn, Fe-Mn-Cu, Fe-Mn-W, and Fe-Mn-Co Alloys

The observed phases, crystallite size, and lattice strain of BM0, BM1, BM2, and BM3 alloys were identified using XRD (Empyrean, Malvern Panalytical, Malvern, UK). Cu-kα source of radiation was applied to examine the samples with 0.6°/min rate and 0.01° step with 2ϴ range of 20° to 90°. The attained data from XRD experiment was studied by X’Pert High Score Plus software (version 2.2b (2.2.2), Furthermore, the same software was used to calculate the lattice strain and crystallite size based on Debye–Scherer concept. Apreo FEG-HR-SEM equipped with SE, BSE, and EDS detectors was used for microstructure analysis of the BM0, BM1, BM2, and BM3 alloys. The powders size and morphology, the elemental dispersion of the processed alloys in both powders and compact forms were characterized. The formation of nanocrystalline nature of four bio-degradable alloys (BM0, BM1, BM2, and BM3) were examined using high-resolution transmission electron microscope (HRTEM, JEOL 3010, Akishima, Tokyo, Japan). Before HRTEM examination, the synthesized powders were dissolved in an ethanol solution, poured over the copper grid, and then placed inside the machine. The used HRTEM was attached with EDAX by which the presence of various elements in BM1 and BM3 alloys, as an example, were also taken.

### 2.3. Compressibility of the Processed Fe-Mn, Fe-Mn-Cu, Fe-Mn-W, and Fe-Mn-Co Alloys

**Figure 1:**
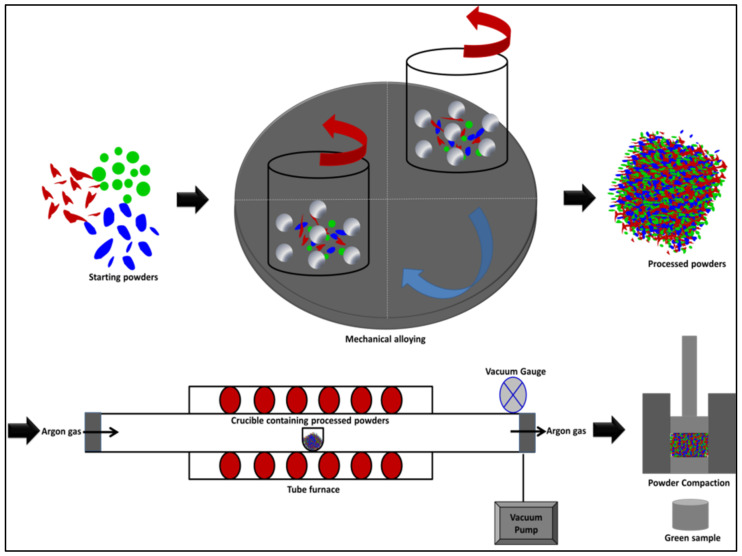
A schematic representation of the powders processing and compaction in this study.

The compressibility of the synthesized alloys was investigated experimentally to study the densification behavior of these alloy powders. The compaction behavior was examined for the processed powders under various loads, ranged from 25 MPa to 1100 MPa, in the as-milled conditions and after applying stress relief treatment. The maximum pressure was decided after several trails where increasing the pressure more than 1100 MPa in the present study resulted in cracking and breaking the green samples. The stress relief treatment was carried out using a tube furnace (model: R120/500/13, Nabertherm, Lilienthal, Germany) where the processed powders were heat treated at 150 °C for one hour under vacuum (−5 psi) and controlled argon atmosphere to prevent powder oxidation. The temperature and time of the heat treatment were selected based on the idea of applying a relatively lower temperature (150 °C) to make use of this treatment in improving the compressibility of the processed powders without causing major changes in the microstructure constituents and features of the alloys that was attained from the mechanical alloying process such as crystallite size and structure homogeneity. The die used in compressibility test was made of hardened H13 tool steel with 15 mm inner diameter and upper/bottom plungers (outer diameter of 15 mm). The compressibility test was accomplished in MTS universal testing machine with 1 mm/min of crosshead speed. The processed powders were compacted at different pressures to generate density–pressure curves. The green compacts density was measured according to Archimedes’ conception where an average of three measurements was recorded. In addition to the experimental study of the powder densification, the compressibility of the as-milled and stress relieved powders was further examined by empirical formulas to determine the optimum empirical equation for expecting the relative density of the fabricated alloys [27,31]. A schematic representation of the powders processing and compaction of the developed nanocrystallite materials is shown in Figure 1. This Figure shows the steps of performing the present study where the starting powders of each alloy system were mechanically alloyed. Thereafter, the obtained processed powders were examined using XRD, HRSEM equipped with EDS, and HRTEM. Specific quantities of the processed powders were heat treated using a tube furnace equipped with a vacuum pump and a controlled atmosphere system. The compressibility experiment was conducted using MTS (Eden Prairie, MN, USA) machine to study the compressibility of the as-milled and the heat treated powders. Ultimately, the relative density and microstructure analysis of the green samples were evaluated.

## 3. Results and Discussion

The present study aims to synthesizing Fe-Mn, Fe-Mn-Cu, Fe-Mn-W, and Fe-Mn-Co nanostructured biodegradable alloys by mechanical alloying; microstructure investigation of the processed alloys using XRD, high resolution SEM equipped with EDS technique, and HRTEM; and studying the compressibility of the processed alloys experimentally and empirically in the as-milled condition and after applying stress relief treatment.

### 3.1. Microstructural Characterization of the Processed Fe-Mn, Fe-Mn-Cu, Fe-Mn-W, and Fe-Mn-Co Alloys

**Figure 2:**
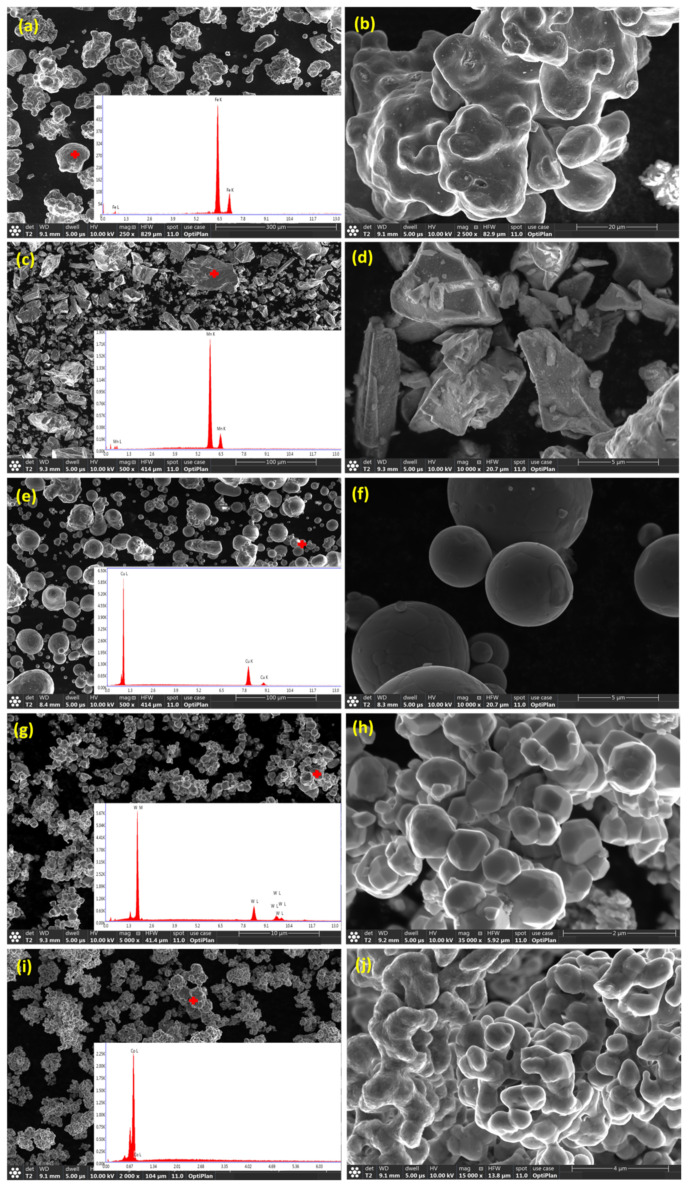
SE images of the as-received powders morphology at low (left) and high magnification (right): (**a**,**b**) Fe particles; (**c**,**d**) Mn powders; (**e**,**f**) Cu powders; (**g**,**h**) W powders; and (**i**,**j**) Co powders. The insets at the low magnification images show the EDS spectrum of the corresponding powders.

**Figure 3:**
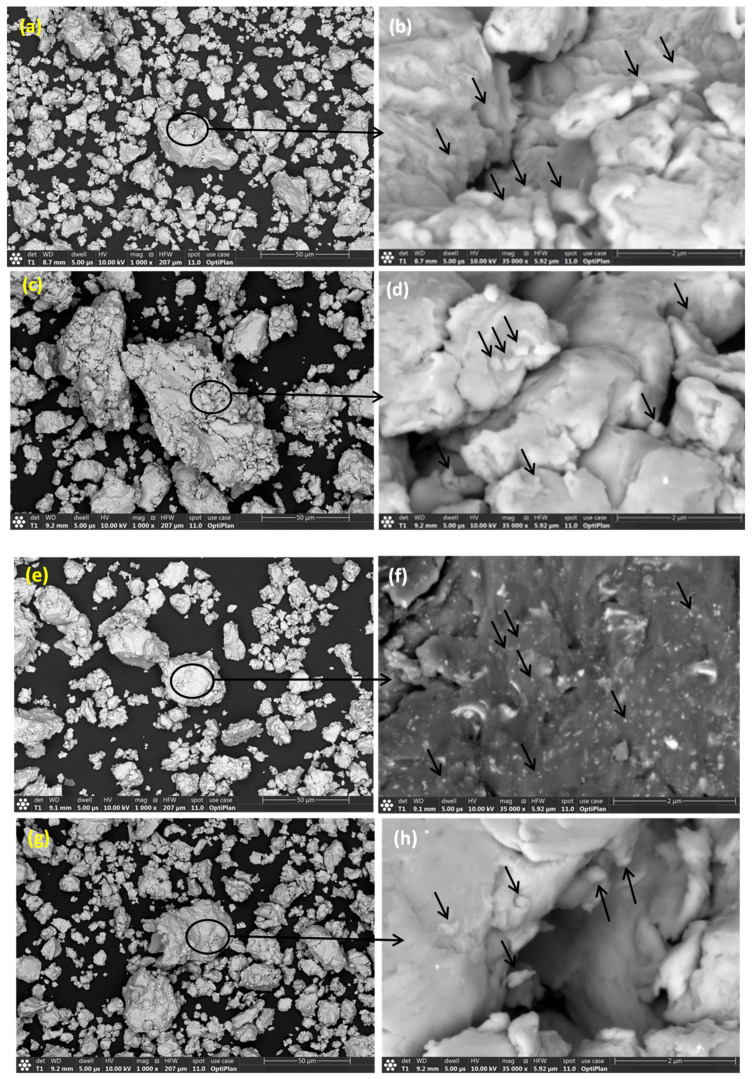
(**a**,**c**,**e**,**g**) BSE images of the morphology of the mAed BM0, BM1, BM2, and BM3 alloys, respectively; (**b**,**d**,**f**,**h**) high magnification BSE images of the circled region in (**a**,**c**,**e**,**g**), respectively.

The average particle size of the as-received powders was ≤44 μm. The morphology of the as-received Fe, Mn, Cu, W, and Co powders was examined using FEG-HR-SEM and the collected images are presented in Figure 2. Figure 2a,b illustrate the dendritic morphology of Fe particles at low and high magnification, respectively. Figure 2c,d show the facet shape of Mn powders at low and high magnification, respectively. Figure 2e,f present the spherical shape of Cu powders at low and high magnification, respectively. Figure 2g,h present the polygonal appearance of W powders at low and high magnification, respectively. Figure 2i,j display the dendritic shape of Co powders at low and high magnification, respectively. The chemical composition of the powders was confirmed by point analysis using EDS where the resultant EDS spectrum corresponding to each powder was inserted in the bottom right-hand corner of the SE images taken at low magnification, as presented in Figure 2. The BSE images presented in Figure 3a,c,e,g illustrate the morphology of the MAed BM0, BM1, BM2, and BM3 alloys in the powder form where an almost rounded-shape of the processed powders was attained. The high magnification BSE images of the circled regions in Figure 3a,c,e,g are presented in Figure 3b,d,f,h to confirm the formation of a homogenous solid solution in the processed BM0, BM1, BM2, and BM3 alloys, where the synthesized powders revealed uniform gray color indicating the even dispersion/dissolution of the mixed elements of the developed alloys. The coarse aggregates shown in Figure 3a,c,e,g consist of fine particulates as a result of the repetitive rupturing and welding of the Maed powders. Examples of the fine particles that form the large agglomerate are arrowed in Figure 3b,d,f,h. This mechanism occurs during the MA process till a homogenous solid solution of the mixed elements was formed.

**Figure 4:**
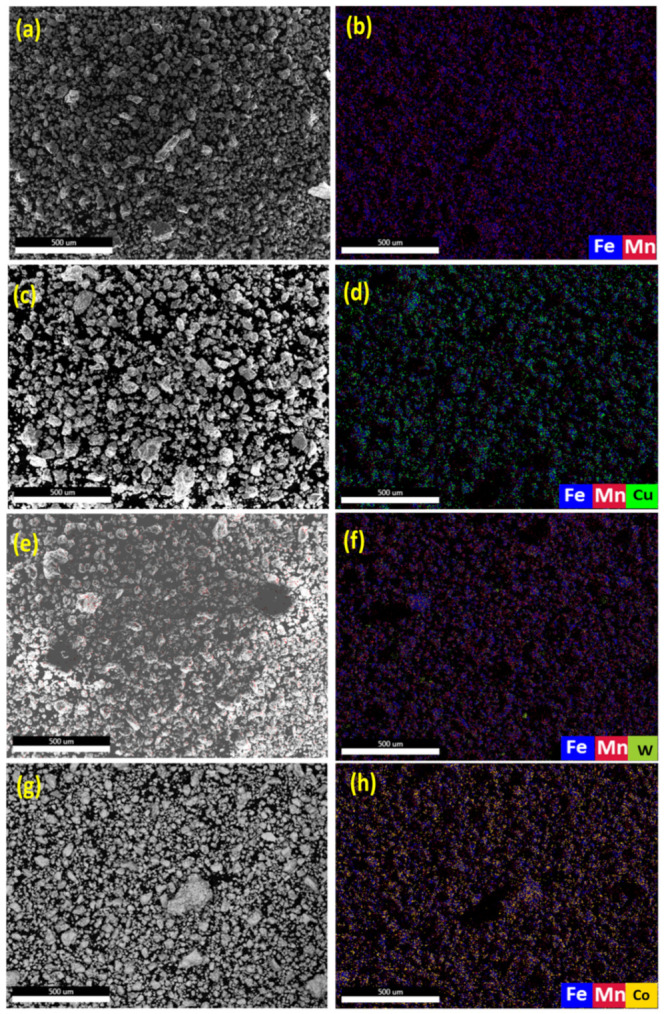
The results of EDS analysis of the MAed BM0, BM1, BM2, and BM3 alloys: (**a**,**c**,**e**,**g**) SE images of BM0, BM1, BM2, and BM3 alloys, respectively; (**b**,**d**,**f**,**h**) overlay maps of the same BM0, BM1, BM2, and BM3 alloys, respectively.

The EDS analysis of the MAed BM0, BM1, BM2, and BM3 alloys are presented in Figure 4. Figure 4a,c,e,g show the SE image of the selected regions for elemental mapping of BM0, BM1, BM2, and BM3 alloys, respectively. Figure 4b,d,f,h display the overlay maps of the MAed BM0, BM1, BM2, and BM3 alloys, respectively. The overlay map in Figure 4b presents the Fe and Mn even dispersion in the MAed BM0 alloy. Figure 4d reveals the uniform dispersion of Fe, Mn and Cu in the MAed BM1 alloy. Figure 4f displays the dispersion of Fe, Mn, and W in the MAed BM2 alloy. Figure 4h shows the dispersion of Fe, Mn, and Co in the MAed BM3 alloy. The results presented in Figure 4 ascertain the development of a homogenous elemental dispersions and solid solution in all the developed alloys.

### 3.2. XRD Analyses of the Developed Fe-Mn, Fe-Mn-Cu, Fe-Mn-W, and Fe-Mn-Co Alloys Powders

**Figure 5:**
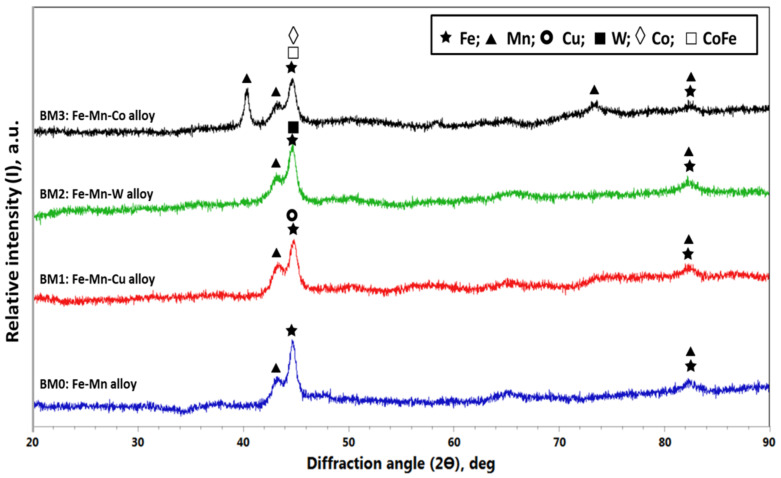
XRD results of MAed BM0, BM1, BM2, and BM3 alloys.

The XRD patterns obtained for BM0, BM1, BM2, and BM3 alloys powders are displayed in Figure 5. For BM0 alloy, the main identified phases were Fe and Mn. With regard to BM1 alloy the same phases (Fe and Mn) were identified in addition to Cu phase. In similar manner, phases of Fe and Mn with the corresponding addition of W and Co were observed in BM2 and BM3 alloys. The characteristics of the observed phases for the developed alloys are displayed in Table 2. The crystallite size and lattice strain were calculated based on the highest peak intensity using X’Pert High Score Plus software according to Debye–Scherrer principles. Regarding Figure 5 and Table 2, the highest intensity peaks in BM0 alloy was 1177.34 cps at 2ϴ of 44.6435 deg; for BM1 alloy the maximum peak intensity was 941.81 cps at 2ϴ of 44.8965 deg; for BM2 alloy the highest peak intensity was 1074.74 cps at 2ϴ of 44.6901 deg; and for BM3 alloy the maximum peak intensity was 884.87 cps at 2ϴ of 40.3947 deg. The calculated crystallite size corresponding to these maximum peaks are 21.4 nm, 21.4 nm, 15.5 nm, and 24.2 nm for BM0, BM1, BM2, and BM3 alloys, respectively. The obtained lattice strain corresponding to the highest peaks are 0.432, 0.432, 0.598, and 0.425 for BM0, BM1, BM2, and BM3 alloys, respectively. These observations assured the effect of mechanical alloying on the reduction of the crystallite size (44 μm to 21.4 nm, for BM0 for example) and the increased lattice strain due to sever plastic deformation during ball milling as noted from the calculated lattice strain (0.432% for BM0, for example).

### 3.3. HRTEM Analyses of Four Synthesized Fe-Mn, Fe-Mn-Cu, Fe-Mn-W, and Fe-Mn-Co Nanostructured Alloys

**Figure 6:**
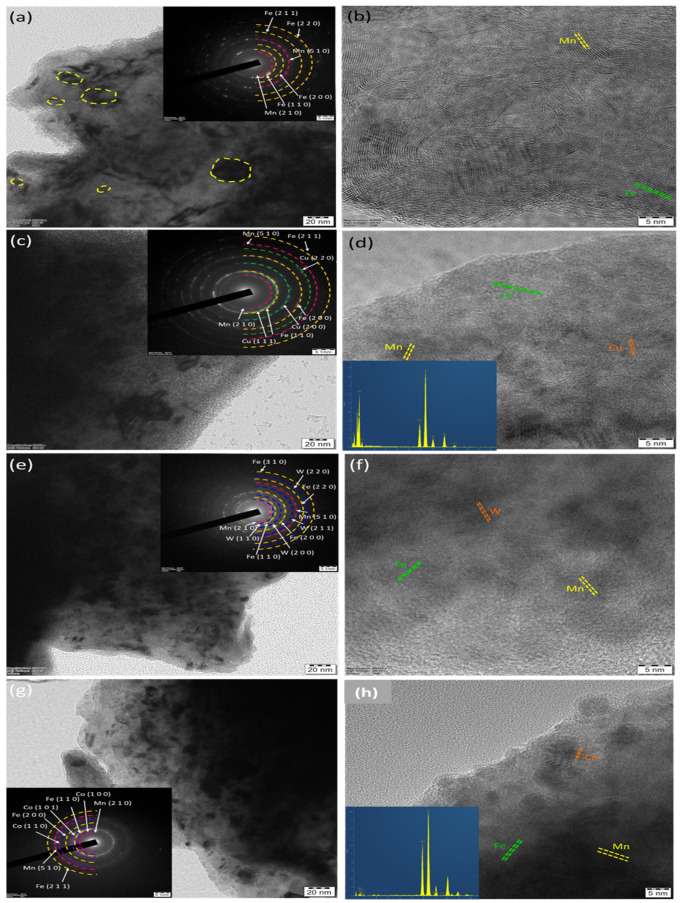
HRTEM analyses of MAed BM0, BM1, BM2, and BM3 nanocrystallite alloy powders: (**a**) bright filed image (BFI) of BM0; (**b**) lattice fringe image (LFI) of BM0; (**c**) BFI of BM1; (**d**) LFI of BM1; € BFI of BM2; (**e**) LFI of BM2; (**f**) BFI of BM3; and (**g**) LFI of BM3. Inset of (**a**,**c**,**e**,**g**) showing the corresponding SAED patterns; inset of (**d**,**h**) showing the corresponding EDS.

The developed alloy powders were characterized by HRTEM and the same is illustrated in Figure 6. From several bright filed and dark field images of each sample, the crystal size was measured using ImageJ software and the average was reported. The average crystallite size of alloy powders was 24.6 ± 3, 22.3 ± 5, 19.5 ± 2, and 25.6 ± 3 nm for BM0, BM1, BM2, and BM3 alloys, respectively. The inset of Figure 6a,c,e,g show the selective area diffraction patterns of corresponding bright filed images. All SAED pattern exhibit the ring pattern conforming the nanostructured formation by the used methodology. Figure 6b,d,f,h show the lattice fringe images of each alloy powder taken at high magnification. The presence of Fe, Mn, Cu, W, and Co lattice was observed based on the corresponding composition of each alloy. For instance, the HRTEM-EDS spectrum was taken for BM1 and BM 4 alloys which exhibited Fe-Mn-Cu and Fe-Mn-Co peaks for BM1 and BM3 alloys, respectively. With regard to the results obtained from XRD, FEG-HR-SEM-EDS, and HRTEM-EDS, it was confirmed that the nanostructured BM0, BM1, BM2, and BM3 alloys were developed successfully using mechanical alloying technique with homogenous solid solutions.

### 3.4. Compressibility of Fe-Mn, Fe-Mn-Cu, Fe-Mn-W, and Fe-Mn-Co Nanostructured Alloys

The compressibility of MAed BM0, BM1, BM2, and BM3 alloys was investigated for the purpose of examining the densification behavior of these alloys. The experiments were conducted at different load up to 1100 MPa. The compressibility of the processed alloys was examined in the as-milled (BM0, BM1, BM2, and BM3) condition and after applying stress relief treatment (150 °C/1 h) to the same alloys (codes for stress relieved samples: BM0-S, BM1-S, BM2-S, and BM3-S). The densification behavior of the same alloys in the as-milled and stress relieved conditions was further studied empirically to identify the optimum model to predict the density of these alloys [31]. A number of linear and non-linear models were applied in the present study for predicting the alloy compressibility. The density of the green samples prepared at 1100 MPa for the as milled and stress relieved samples are listed in Table 3.

The attained relative density of the as milled BM0, BM1, BM2, and BM3 alloys was 72.89%, 71.64%, 72.38%, and 72.03%, respectively. After applying the stress relief treatment, the relative density BM0-S, BM1-S, BM2-S, and BM3-S alloys samples was observed to increase to 75.23%, 77.10%, 72.65%, and 72.86%, respectively. The enhanced relative density as a result of applying stress relief treatment is related to the decreased resistance to powder compressibility due to the reduced lattice strain and diminished strain hardening of the heat-treated powders. However, the observed improvements in relative density of the samples studied are limited due to low temperature and short time applied for heat treatment. This heat treatment was proposed to reduce lattice strain with minimizing the probability of grain growth and structural changes.

**Figure 7:**
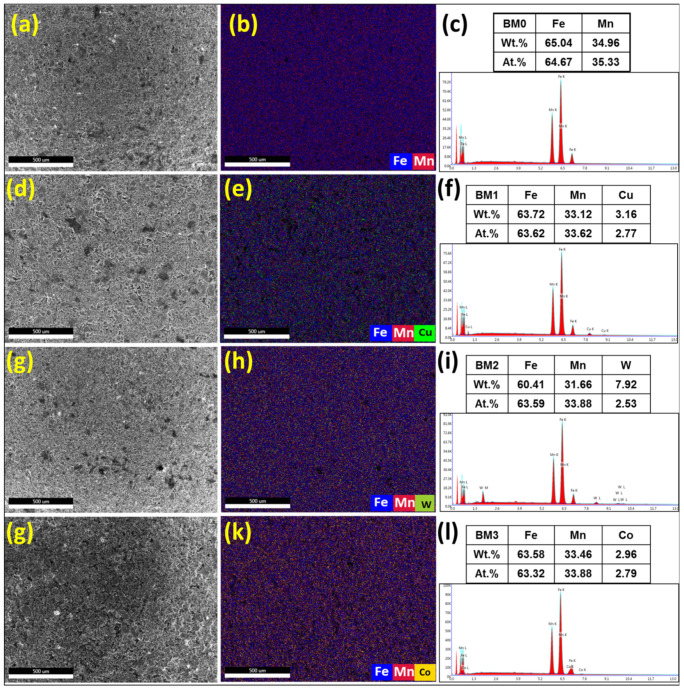
Secondary electron images, overlay elemental dispersion and EDS composition analysis results of the compacted samples: (**a**–**c**) the results for BM0 alloy; (**d**–**f**) the results for BM1 alloy; (**g**–**i**) the results for BM2 alloy; and (**j**–**l**) the results for BM3 alloy.

The microstructure and elemental dispersion in BM0, BM1, BM2, and BM3 green samples are displayed in Figure 7. Figure 7a–c display the SE image, overlay elemental dispersion and EDS composition analysis, respectively for BM0 compacted sample. Figure 7d–f reveal the SE image, overlay map and EDS results, respectively for BM1 green sample. Figure 7g–i show the SE image, overlay elemental distribution and EDS analysis, respectively for BM2 compressed sample. Figure 7j–l present the SE image, overlap elemental dispersion and EDS results, respectively for BM3 green sample. The EDS results of elemental mapping represented in Figure 7 confirmed the formation of homogenous solid solution of Mn in Fe in the case of BM0 compacted sample, and uniform dispersion of Cu, W, and Co in the matrix phase corresponding to BM1, BM2, and BM3 green samples, respectively.

**Figure 8:**
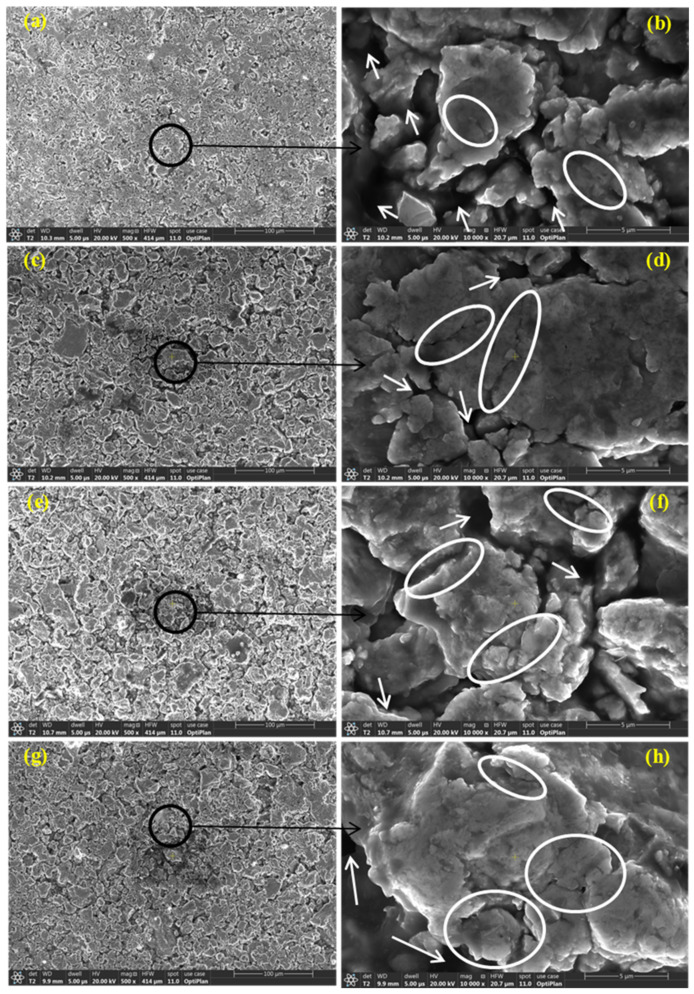
Secondary electron images at low and high magnifications displaying the overview of the green samples: (**a**,**b**) BM0 alloy; (**c**,**d**) BM1 alloy; (**e**,**f**) BM2 alloy; and (**g**,**h**) BM3 alloy.

Figure 8a,c,e,f reveal SE images at low magnifications displaying the overview of BM0, BM1, BM2, and BM3 green sample, respectively. Figure 8b,d,g,h show SE images at high magnifications of BM0, BM1, BM2, and BM3 compacted sample, respectively. The arrows refer to the voids present in the green compact while the circled regions refer to the coalescence of the particle, as a result of compaction process. The voids remained without complete welding/coalescence of powders due to the high lattice strain and strain hardening accompanying the MA process, which reduced the compressibility of the MAed powders.

**Figure 9:**
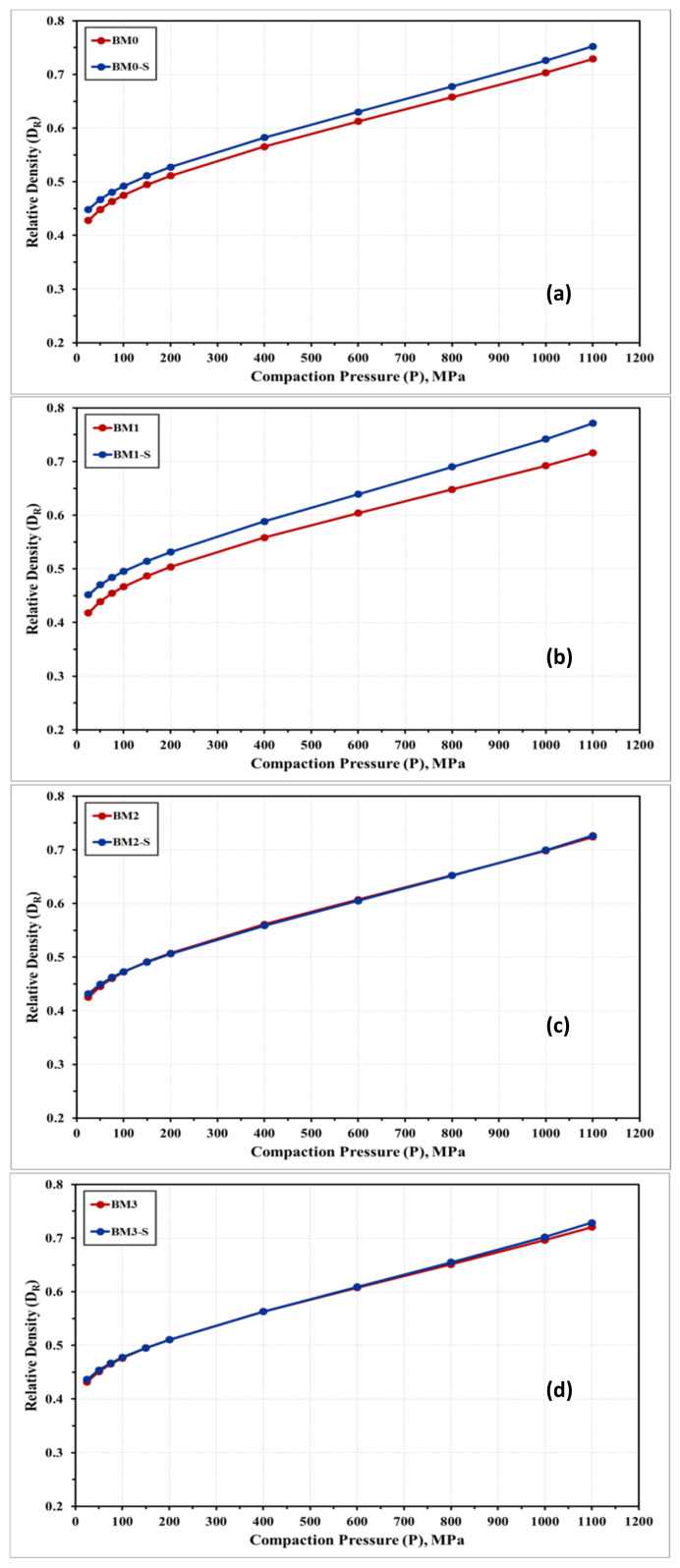
Relative density of (**a**) BM0; (**b**) BM1; (**c**) BM2; and (**d**) BM3 samples in the as-milled vs. stress relieved conditions.

Figure 9 shows the compressibility curves obtained experimentally for the alloys under investigation in the as-milled and stress relieved conditions. The densification behavior of BM0, BM0-S, BM1, BM1-S, BM2, BM2-S, BM3, and BM3-S samples are almost similar with a notable improved compressibility response for the stress relieved samples in BM0 and BM1 samples, as illustrated in Figure 9a,b. Increasing the compaction load resulted in a considerable increase in the relative density of synthesized alloys. The compressibility behavior in Figure 9 exhibited three steps of compaction. Firstly, a rapid increase in the alloy density in the range of 25–100 MPa due to the reordering of powder particles, which resulted in reducing the voids among these powders and decreasing the powders volume with achieving approximately 50% relative density for BM1-S alloy, for instance. Secondly, a further increase in the relative density was recorded in the range of 100–200 MPa. The major mechanism in this second step is the powder deformation, which increased the powders contact and reduced the volume of voids. This mechanism results in reducing the volume of the compacted sample and consequently an additional increase in the sample relative density to 53% was attained for BM1-S sample, as an example. The curve in the second step was noted to display a lower slope as compared to step one. Finally, in the range of 200–1100 MPa, the curve exhibited a further increase in relative density and the maximum value (77% for BM1-S alloy) was attained at 1100 MPa; however, the slope of the curve in this phase is lesser than that in the second step. The governing mechanism in the third stage is the powders impingement under the applied load. From Table 3 and Figure 9a,b it was noticed that the relative density of BM0 and BM1 alloys were enhanced by applying stress relief treatment (BM0-S and BM1-S samples). Heating the processed powders resulted in improving the powder compressibility, which was attributed to the effect of stress relief treatment on reducing the lattice strain and increasing the deformability of powders that led to reduced level of voids and increased contact among powder particles. On the other hand, for BM2 and BM3 alloys the effect of stress relief treatment was not significant. When adding W (BM2) or Co (BM3), the temperature and/or the time of the stress relief treatment should be increased for effective stress recovery to display significant effect on the compressibility of these alloys.

The compressibility behavior of the Fe-Mn, Fe-Mn-Cu, Fe-Mn-W, and Fe-Mn-Co alloys was additionally examined empirically by a number of linear and non-linear models. These models correlate the attained density with the loads applied during compaction. Based on the obtained experimental results, these models will be used for expecting the green density of the produced nanocrystalline alloys in the as-milled and stress relieved conditions. The models applied in the present study are described below where the linear models are presented in Equations (1) through (5) while the nonlinear ones are displayed in Equations (6) through (8).

Balshin’s model [32]:

(1) $$\frac{1}{D_{R}}=KlnP+A$$

Heckel’s model [33]:

(2) $$ln\left(\frac{1}{1-D_{R}}\right)=KP+A$$

Ge’s model [34]:

(3) $$log\left[ln\frac{1}{1-D_{R}}\right]=KlogP+A$$

Panelli and Ambrosio Filho’s model [35]:

(4) $$ln\left(\frac{1}{1-D_{R}}\right)=K\sqrt{P}+A$$

Kawakita’s model [36]:

(5) $$\frac{D_{R}}{D_{R}-D_{o}}=\frac{K}{P}+A$$

Shapiro’s model [37]:

(6) $$ln\left(1-D_{R}\right)=ln\left(1-D_{o}\right)-CP-B\sqrt{P\;}+A$$

Cooper and Eaton’s model [38]:

(7) $$\frac{D_{R}-D_{o}}{D_{R}\left(1-D_{o}\right)}=a_{1}exp\left(\frac{-k_{1}}{P}\right)+a_{2}exp\left(\frac{-k_{2}}{P}\right)$$

Van Der Zwan and Siskens’s model [39]:

(8) $$\frac{D_{R}-D_{o}}{\left(1-D_{o}\right)}=a\;exp\left(\frac{-k}{P}\right)$$

**Figure 10:**
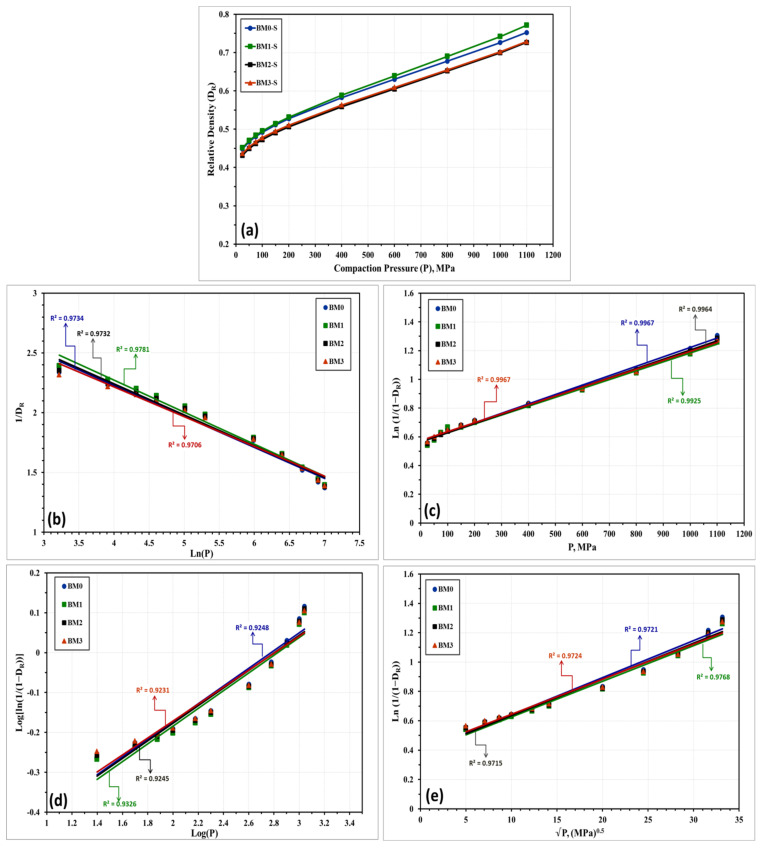

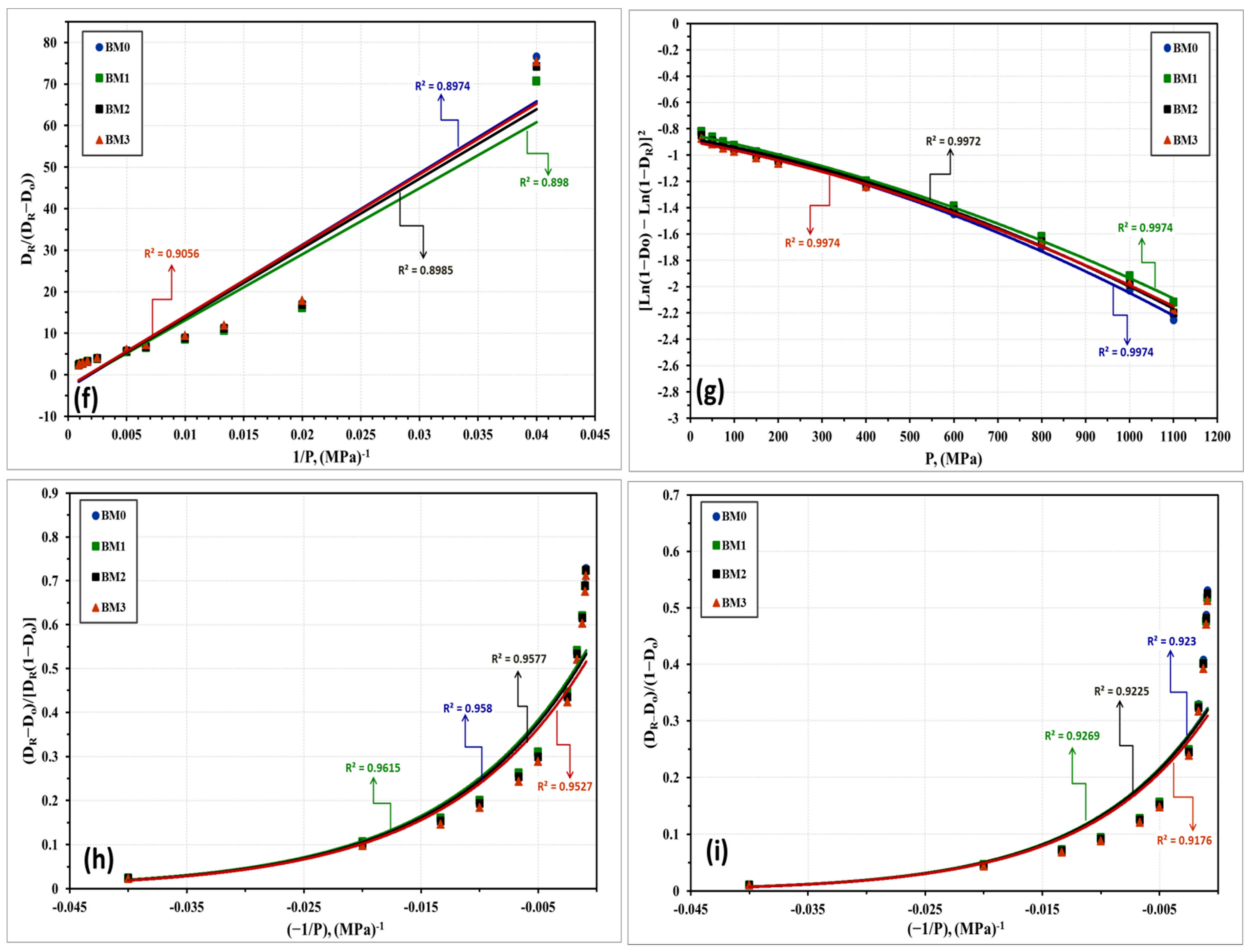
Compressibility results of the as-milled BM0, BM1, BM2, and BM3 alloy samples obtained from: (**a**) compressibility experiments conducted on these alloys. (**b**–**i**) are the compressibility results obtained empirically from applying models (1) through (8), one-to-one.

**Figure 11:**
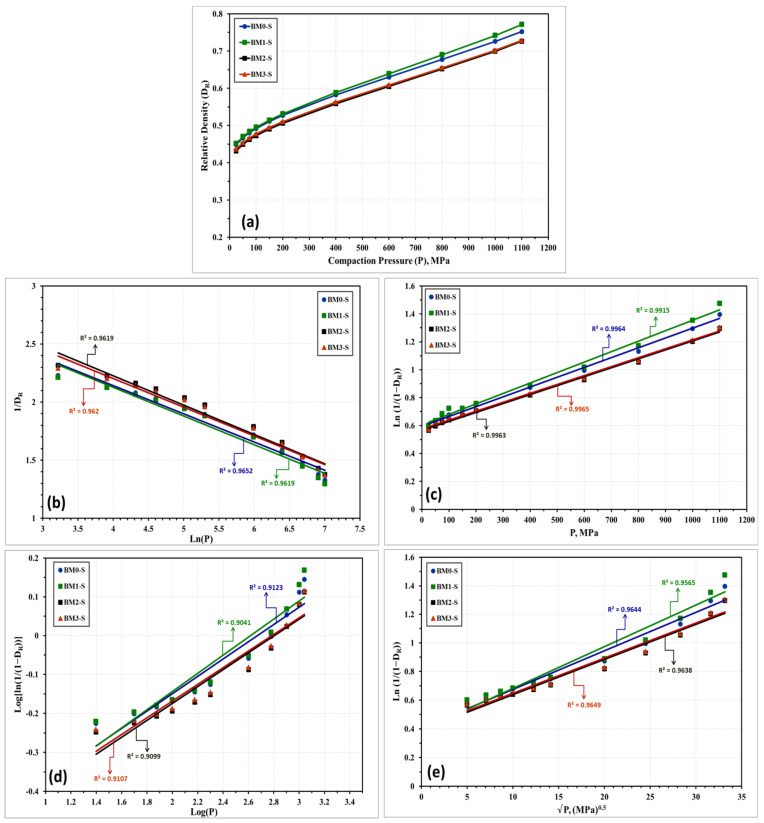

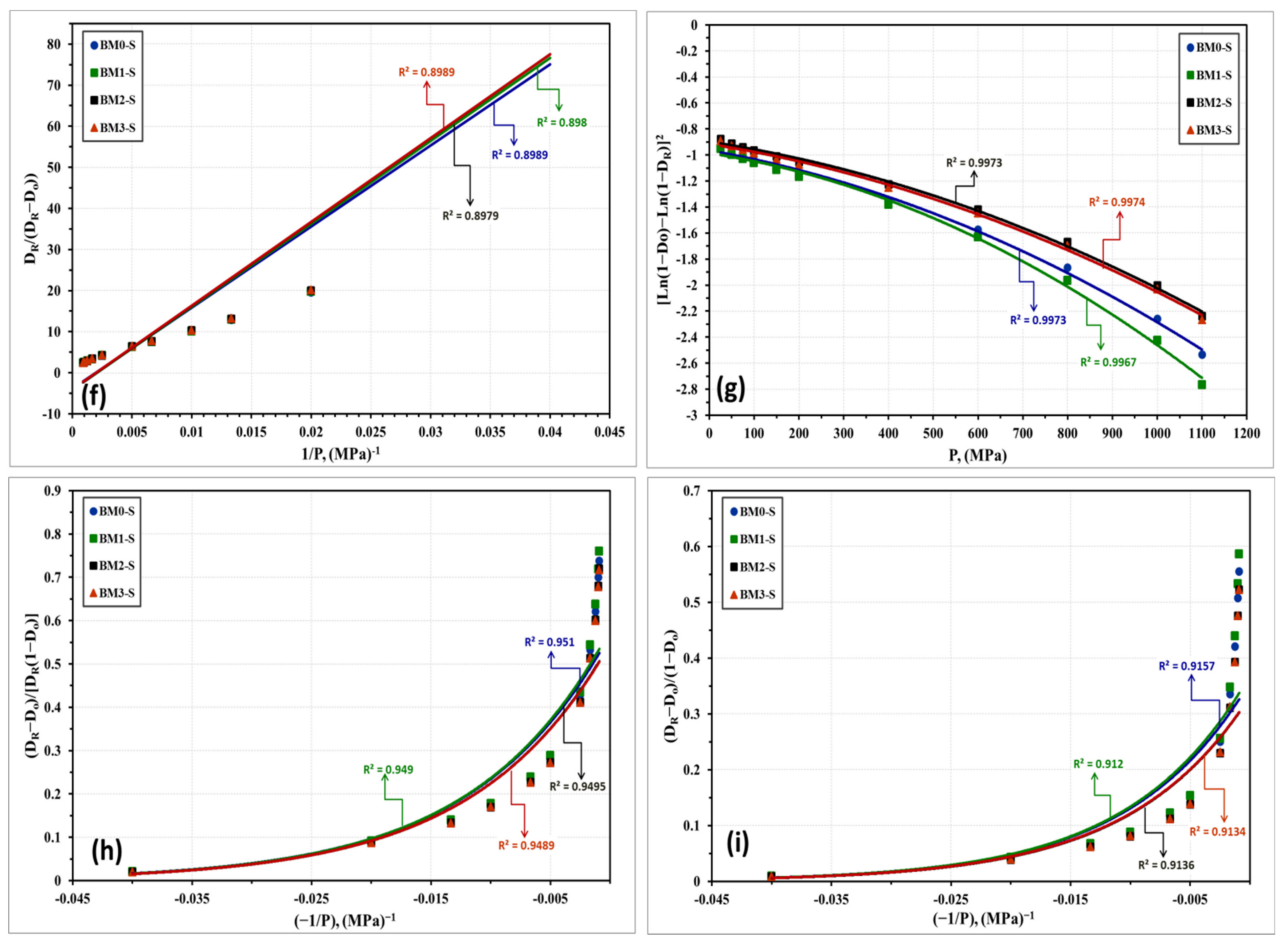
Compressibility results of the stress-relieved samples BM0-S, BM1-S, BM2-S, and BM3-S alloy samples obtained from: (**a**) compressibility experiments conducted on these alloys. (**b**–**i**) the compressibility results obtained empirically from applying models (1) through (8), one-to-one.

With regard to the above-listed models: “*P*” is the applied load; “*D_R_*” is relative density; “*D_o_*” is the relative apparent density; and *A*, *a*, *a*_1_, *a*_2_, *K*, *k*_1_, and *k*_2_ are specific factors for each model that can be estimated from the data fitting of the compressibility curves. Figure 10 and Figure 11 represent the compressibility behavior of BM0, BM1, BM2, and BM3 alloys in the as-milled and stress relieved conditions, respectively. Figure 10a and Figure 11a display the compressibility of the as-milled (BM0, BM1, BM2, and BM3) and stress relieved (BM0-S, BM1-S, BM2-S, and BM1-S) samples based on the experimentations performed in the present study. Figure 10b–i show the compressibility behavior of the as-milled samples based on models 1 through 8, respectively, while Figure 11b–i display the compaction curves of the stress-relieved samples according to equations 1 through 8, respectively. The estimated factors related to each model based on the experimentations performed in the current study are presented in Table 4. The compressibility results displayed in Table 4 and Figure 10 and Figure 11 illustrate that the general behavior of BM0/BM0-S, BM1/BM1-S, BM2/BM2-S, and BM3/BM3-S alloys are almost similar with the exception of the positive influence of the stress-relief treatment on compressibility of BM0-S and BM1-S samples. From the analysis of these results, the optimum models for predicating the compressibility of the alloys under study in the as-milled and stress-relieved conditions are Equation (2) (linear) and Equation (6) (non-linear). The application of Equation (2) and Equation (6) revealed the highest R^2^-values of 0.9967 and 0.9974, respectively (in the case of BM0 alloy, for instance). In contrast, Equation (5) (linear) and Equation (8) (non-linear) exhibited the lowest R^2^-values of 0.8974 and 0.9230, respectively (for BM0 alloy, for example). Consequently, Heckel’s linear model presented in Equation (2) and the Shapiro’s non-linear model displayed in Equation (6) are the optimum compaction models for predicting the compressibility behavior for nanostructured Fe-Mn, Fe-Mn-Cu, Fe-Mn-W, and Fe-Mn-Co alloys in the as-milled and stress-relieved conditions.

## 4. Conclusions

- The nanostructured Fe-Mn (BM0), Fe-Mn-Cu (BM1), Fe-Mn-W (BM2), and Fe-Mn-Co (BM3) alloys were successfully synthesized in the present study using mechanical alloying.
- The XRD, SEM-EDS, and HRTEM results confirmed the formation of homogenous solid solutions with an even elemental dispersion of alloys with the attainment of nanostructured alloys.
- The attained relative density of the as-milled BM0, BM1, BM2, and BM3 alloys at 1100 MPa was 72.90%, 71.64%, 72.38%, and 72.03%, respectively. After applying the stress relief treatment, the density was observed to increase to 75.23%, 77.10%, 72.65%, and 72.86% for BM0-S, BM1-S, BM2-S, and BM30S samples at 1100 MPa, respectively.
- The optimum models for predicating the compressibility of the alloys under study in the as-milled and stress-relieved conditions were Heckel’s linear model and Shapiro’s non-linear model which revealed the highest R2-values of 0.9967 and 0.9974, respectively (in the case of BM0 alloy for instance).

## Figures and Tables

**Table 1:** Composition of the produced Fe-Mn, Fe-Mn-Cu, Fe-Mn-W, and Fe-Mn-Co alloys.

Alloy Code	Composition in at.% (Wt.%)				
	Fe	Mn	Cu	W	Co
BM0 *	65.00 (65.36)	35.00 (34.64)	---	---	---
BM1 *	65.00 (65.07)	32.00 (31.51)	3.00 (3.42)	---	---
BM2 *	65.00 (61.12)	32.00 (29.60)	---	3.00 (9.29)	---
BM3 *	65.00 (65.23)	32.00 (31.59)	---	---	3.00 (3.18)

* BM0-S, BM1-S, BM2-S, and BM3-S codes will be used instead of BM0, BM1, BM2, and BM3, respectively, for the same alloys subjected to stress relief treatment.

**Table 2:** The characteristics of the observed phases in MAed BM0, BM1, BM2, and BM3 alloys.

Alloy Code	Observed Phase	Position 2ϴ, Deg	Peak Height, Cps	Code	Structure	Crystal Size (nm)	Lattice Strain (%)
BM0	Fe	44.6435	1177.34	01-085-1410	Cubic	21.4	0.432
		82.3443	104.51	01-085-1410	Cubic		
	Mn	42.9402	277.57	00-001-1237	Cubic		
		82.3443	104.51	00-003-1014	Cubic		
BM1	Fe	44.8965	941.81	01-087-0722	Cubic	21.4	0.432
		82.2603	92.06	01-087-0722	Cubic		
	Mn	43.3474	293.13	00-001-1234	Cubic		
		82.2603	92.06	00-001-1234	Cubic		
	Cu	43.3474	293.13	01-089-2838	Cubic		
BM2	Fe	44.6901	1074.74	01-087-0721	Cubic	15.5	0.598
		82.1962	213.56	01-087-0721	Cubic		
	Mn	43.7106	212.34	00-001-1234	Cubic		
		82.1962	213.56	00-001-1234	Cubic		
	W	44.6901	1074.74	01-088-2339	Cubic		
BM3	Fe	44.7608	862.36	01-087-0722	Cubic	24.2	0.425
		82.6692	133.67	01-087-0722	Cubic		
	Co	44.7608	862.36	00-001-1254	Cubic		
	CoFe	44.7608	862.36	00-044-1433	Cubic		
	Mn	40.3947	884.87	00-032-0637	Cubic		
		42.9173	161.46	00-032-0637	Cubic		
		73.5610	96.49	00-032-0637	Cubic		
		82.6692	133.67	00-032-0637	Cubic		

**Table 3:** The density of the green samples prepared at 1100 MPa for the as milled and stress relieved samples.

Alloy Code	Theoretical Density (g/cm^3^)	Green Density (g/cm^3^)	Relative Density (%)
BM0	7.73	5.63	72.90
BM0-S	7.73	5.81	75.23
BM1	7.77	5.77	71.64
BM1-S	7.77	5.99	77.10
BM2	8.19	5.93	72.38
BM2-S	8.19	5.95	72.65
BM3	7.77	5.59	72.03
BM3-S	7.77	5.66	72.86

**Table 4:** The estimated factors related to the models applied on the alloys investigated in the present study.

Applied Model	Factor	Alloy Code							
		BM0	BM0-S	BM1	BM1-S	BM2	BM2-S	BM3	BM3-S
Balshin [32]	A	3.2633	3.1128	3.3414	3.1163	3.2822	3.2384	3.2107	3.1931
	K	−0.2950	−0.2430	−0.2670	−0.2470	−0.2600	−0.2530	−0.2490	−0.247
	R^2^	0.9734	0.9652	0.9781	0.9619	0.9732	0.9619	0.9706	0.9620
Heckel [33]	A	0.5685	0.5961	0.5665	0.6054	0.5639	0.5659	0.5751	0.5749
	K	0.0007	0.0007	0.0006	0.0007	0.0006	0.0006	0.0006	0.0006
	R^2^	0.9967	0.9964	0.9925	0.9915	0.9964	0.9963	0.9967	0.9965
Ge [34]	A	−0.6171	−0.5949	−0.6280	−0.6099	−0.6176	−0.6072	−0.5969	−0.5972
	K	0.2223	0.2228	0.2220	0.2332	0.2204	0.2165	0.2128	0.2144
	R^2^	0.9248	0.9123	0.9326	0.9041	0.9245	0.9099	0.9231	0.9107
Panelli and Ambrosio Filho’s [35]	A	0.3895	0.4057	0.3825	0.3900	0.3885	0.3924	0.4050	0.4013
	K	0.0252	0.0270	0.0243	0.0292	0.0247	0.0246	0.0239	0.0246
	R^2^	0.9721	0.9644	0.9768	0.9565	0.9715	0.9638	0.9724	0.9649
Kawakita [36]	A	−3.1733	−3.9202	−2.7487	−4.1445	−2.9476	−4.1586	−2.8135	−4.0914
	K	1725	1976.1	1590.6	2019.6	1671.3	2041.7	1701.7	2040.8
	R^2^	0.8974	0.8989	0.8980	0.8980	0.8985	0.8979	0.9056	0.8989
Shapiro [37]	A	−0.8826	−0.9652	−0.8428	−0.9865	−0.8725	−0.8984	−0.8951	−0.9154
	B	−6 × 10^−4^	−6 × 10^−4^	−7 × 10^−4^	−5 × 10^−4^	−6 × 10^−4^	−5 × 10^−4^	−6 × 10^−4^	−5 × 10^−4^
	C	−5 × 10^−7^	−7 × 10^−7^	−4 × 10^−7^	−1 × 10^−6^	−5 × 10^−7^	−6 × 10^−7^	−5 × 10^−7^	−6 × 10^−7^
	R^2^	0.9974	0.9973	0.9974	0.9967	0.9972	0.9973	0.9974	0.9974
Cooper and Eaton [38]	a_1_	0.4194	0.4083	0.4291	0.4115	0.4165	0.3899	0.7308	0.3868
	a_2_	0.7248	0.7866	0.6836	0.8324	0.7330	0.7959	0.4034	0.7886
	k_1_	79.6217	86.1326	77.3395	86.8809	77.8816	86.1326	909.090	85.470
	k_2_	884.955	909.091	869.565	917.4312	900.9	925.925	80.9716	917.43
	R^2^	0.9580	0.9510	0.9615	0.9490	0.9577	0.9495	0.9527	0.9489
Van Der Zwan and Siskens [39]	A	0.3524	0.3574	0.3505	0.3698	0.3477	0.3316	0.3370	0.3324
	K	97.9340	100.2600	96.5250	101.6200	97.0280	100.9900	96.3640	100.5400
	R^2^	0.9230	0.9157	0.9269	0.9120	0.9225	0.9136	0.9176	0.9134

## Data Availability

The experimental datasets obtained from this research work and then the analyzed results during the current study are available from the corresponding author on reasonable request.
